# Diagnostic accuracy of phosphatase and tensin homolog loss in differentiating between atypical endometrial hyperplasia/endometrioid intraepithelial neoplasia and non‐atypical endometrial hyperplasia: A systematic review and meta‐analysis

**DOI:** 10.1002/ijgo.70901

**Published:** 2026-02-26

**Authors:** Kristina Shcherbatiuk, Steffi Bultmann, Thomas Karn, Khayal Gasimli, Peter Wild, Sven Becker, Morva Tahmasbi Rad

**Affiliations:** ^1^ Department of Gynaecology and Obstetrics Johann Wolfgang Goethe University Frankfurt am Main Germany; ^2^ Department of Pathology Johann Wolfgang Goethe University Frankfurt am Main Germany

**Keywords:** diagnostic tests, endometrial hyperplasia, immunohistochemistry, meta‐analysis, PTEN phosphohydrolase, systematic review

## Abstract

**Background:**

Accurate distinction between atypical endometrial hyperplasia/endometrioid intraepithelial neoplasia (AH/EIN) and non‐atypical endometrial hyperplasia (EH) determines whether patients require hysterectomy or can be managed conservatively. Phosphatase and tensin homolog (PTEN) loss has been proposed as an immunohistochemical (IHC) marker to support diagnostic evaluation.

**Objectives:**

This study evaluates the diagnostic value of PTEN IHC for the diagnosis of AH/EIN and to explore how PTEN assessment methods affect diagnostic performance.

**Method:**

PubMed, Cochrane Library, ClinicalTrials.gov, Scopus, Embase, MEDLINE, and Web of Science were searched from database inception up to June 1, 2025. Studies reporting PTEN IHC in women with histologically confirmed AH/EIN or non‐atypical EH. Two reviewers independently screened records, extracted data, and assessed risk of bias with QUADAS‐2. Pooled diagnostic estimates and summary receiver‐operating characteristics (SROC) curves were calculated using a bivariate random‐effects model with subgroup analysis and the IHC method.

**Results:**

Twenty‐seven studies (2274 women) were included. Pooled sensitivity was 58.0% (95% confidence interval [CI], 40.8–73.4) and specificity 84.7% (95% CI, 67–93.8). The diagnostic odds ratio was 3.695 (95% CI, 2.501–5.460), with an area under the SROC of 0.679. The PTEN assessment method based on percentage of stained cells and staining intensity showed the highest the area under the curve of 0.744 among the methods evaluated in the subgroup analysis.

**Conclusions:**

PTEN IHC assessment demonstrated good specificity and might serve as a complementary tool in diagnosing AH/EIN, helping to reduce the number of incorrect diagnoses of atypia and unnecessary hysterectomies in clinical practice. Its limited sensitivity precludes its use as a standalone marker. Standardized staining protocols and multi‐marker strategies are needed.

**PROSPERO Registration:**

CRD42024607623, registration date the 29 October 2024.

## 1 INTRODUCTION

Endometrial cancer (EC) is the sixth most common cancer in terms of both incidence and mortality in women, with an estimated 421 000 new cases and 98 000 deaths worldwide by 2022.^1^ Atypical hyperplasia/endometrioid intraepithelial neoplasia (AH/EIN) is recognized as the precursor to endometrioid‐type EC, with an estimated 27.5% risk of progression over 20 years, compared to less than 4.6% for non‐atypical endometrial hyperplasia (EH).^2^ Accurate distinction between benign and premalignant conditions is crucial for patient management. Despite the effectiveness of medical therapy for EH, total hysterectomy remains the standard of care for patients with AH/EIN or grade 1 EC.^3^, ^4^ Given the rising incidence of young women diagnosed with precancerous lesions or early‐stage EC, accurate diagnosis is critical to enable fertility‐sparing treatment.^5^, ^6^ For elderly patients with significant comorbidities, avoiding unnecessary surgery is equally important.

Histological examination is considered the gold standard in differentiating premalignant from benign EH. The World Health Organization (WHO, 1994) classification stratified EH into four categories based on glandular complexity and cytologic atypia. Later, the EIN system classified EH by incorporating morphologic characteristics, molecular alterations, and computerized morphometry.^7^ In 2014, the WHO (WHO, 2014) consolidated the classification into two categories (EH without atypia and atypical EH), merging the AH and EIN terminology.^8^ However, diagnosis remains challenging due to interobserver variability, specimen fragmentation, tissue inadequacy, and treatment‐related cytologic changes.^9^ To address these issues, the 2020 WHO Classification of Female Genital Tumors retained the WHO 2014 criteria and recommended supplementing histology with immunohistochemical (IHC) markers. Loss of PTEN, PAX2, or mismatch repair proteins is identified as a desirable diagnostic criterion for AH/EIN.^10^ However, standardized criteria for PTEN IHC assessment and interpretation remain lacking, limiting its clinical implementation.

Phosphatase and tensin homolog (PTEN) is a tumor suppressor and the most frequently mutated gene in EC and AH/EIN.^11^, ^12^ PTEN negatively regulates the PI3K/AKT/mTOR signaling pathway, thereby inhibiting endometrial cell proliferation.^13^ PTEN inactivation through mutation or epigenetic silencing is considered an early driver event in endometrial carcinogenesis.^12^, ^14^, ^15^

The PTEN loss detected by IHC reflects neoplastic changes and might help differentiate AH/EIN from benign hyperplasia in morphologically ambiguous cases supporting clinical management. However, studies applying WHO 2014/EIN diagnostic criteria report PTEN loss in 40.7%–70% of AH/EIN cases, indicating substantial variability across the literature.^16^, ^17^

In addition, PTEN IHC assessment methods differ across studies. Evaluation might be based on staining intensity, ranging from absent to strong; the percentage of positively or negatively stained cells with a defined cutoff; identification of PTEN‐null glands; or combined evaluation of staining percentage and intensity. These methodological differences might lead to inconsistent interpretation of PTEN loss and influence diagnostic performance.

This systematic review and meta‐analysis aims to assess the diagnostic value of PTEN loss in distinguishing AH/EIN from non‐atypical EH and to evaluate how PTEN assessment methods affect its diagnostic performance, with the goal of informing clinical practice.

## 2 METHODS

This systematic review was conducted in accordance with the Preferred Reporting Items for Systematic Reviews and Meta‐Analyses of Diagnostic Test Accuracy (PRISMA‐DTA) guidelines and was registered with the International Prospective Register of Systematic Reviews (PROSPERO) on October 29, 2024 (CRD42024607623).^18^

### 2.1 Information sources

An electronic database search was performed in PubMed, Cochrane Library, ClinicalTrials.gov, Scopus, Embase, MEDLINE, and Web of Science from database inception until June 4, 2024, and was updated on June 1, 2025. No restrictions were applied to language or publication period during the search stage. The keywords used in our search strategy included: ((endometrial hyperplasia) OR (endometrial intraepithelial neoplasia) OR (EIN)) AND ((PTEN) OR (phosphatase and tensin homolog)) AND ((marker) OR (biomarker)) AND ((immunohistochemistry) OR (immunohistochemical)) AND (diagnosis). The full search strategy is provided in Table S1. Reference lists of eligible articles were manually screened to identify additional relevant studies.

### 2.2 Eligibility criteria

Studies were eligible if they included women with histologically confirmed AH/EIN and non‐atypical EH and evaluated PTEN expression in endometrial tissue by immunohistochemistry (IHC). Trials that involved both AH/EIN and EH without atypiaand provided sufficient information to calculate, true positives (TP), true negatives (TN), false positives (FP) and false negatives (FN) were included. Non‐atypical EH (benign) was defined as simple or complex EH without atypia, benign EH, low‐risk non‐EIN lesions (*D*‐score >1), and benign proliferative changes.

Hyperproliferative endometrial changes related to unopposed estrogen exposure (including benign architectural changes of unopposed estrogen, disordered proliferative endometrium, and persistent proliferative endometrium) were also classified as non‐atypical EH because they represent a spectrum within the benign hyperplasia sequence associated with excess estrogen.^19^, ^20^ Atypical EH (precancer) included simple or complex atypical EH, intermediate/high‐risk EIN lesions (*D*‐score ≤1), and AH/EIN. If multiple reports from the same studies were identified, the most methodologically comprehensive publication was included to prevent data duplication and reduce potential risk of bias. We excluded studies that did not assess the diagnostic accuracy of PTEN relative to histological diagnosis, as well as reviews, conference abstracts, letters to the editor, editorials, study protocols, case reports, and articles not published in English.

### 2.3 Study selection

Study selection was conducted using Covidence software (Veritas Health Innovation). Two investigators (K.S. and M.T.R.) independently screened titles and abstracts for inclusion, and then assessed full texts for eligibility. Decisions regarding study inclusion were based on predefined eligibility criteria, and full‐text studies were included only when all necessary diagnostic accuracy data (TP, FP, FN, and TN) were clearly extractable. Disagreements regarding final study inclusion were resolved by consensus.

### 2.4 Data extraction

Data extracted included: first author, publication year, country, number of patients, age, EH classification used (WHO 1994, EIN, WHO 2014), diagnostic outcomes, sampling methods. We also recorded details of PTEN assessment methods (staining intensity, percentage of cells staining, combination of percentage and intensity, presence of PTEN‐null glands), definitions of PTEN loss, and interpretation criteria. PTEN loss was defined using the criteria applied by each individual study. PTEN loss was defined as the lowest category of PTEN expression reported by the authors, based on percentage thresholds, staining intensity scales, combined evaluation of percentage and intensity (including both integration into scoring systems and qualitative assessment), or the presence of any PTEN‐null glands. The PTEN assessment method identified by the authors as the primary or most diagnostically relevant was extracted for data synthesis. We extracted data on TP, FP, FN, and TN (regarding the diagnostic value of PTEN loss in identifying AH/EIN) and whether PTEN immunostaining interpretation was conducted by a single pathologist, multiple independent observers, or by consensus. If data were missing, study authors were contacted by email. When the missing information was not received, studies were excluded from the quantitative synthesis. Disagreements regarding data extraction were resolved through consensus with the co‐authors.

### 2.5 Assessment of risk of bias

The risk of bias of included studies was assessed according to the Quality Assessment Tool for Diagnostic Accuracy Studies (QUADAS‐2), independently by two reviewers.^21^ The index test was defined as loss of PTEN expression, and histopathological diagnosis served as the reference standard.

### 2.6 Data synthesis

Pooled sensitivity, specificity, positive likelihood ratios (PLR), negative likelihood ratios (NLR), and diagnostic odds ratio (DOR) were calculated. Meta‐analysis was conducted using a bivariate logistic regression model with random effects (bivariate generalized linear mixed model). The bivariate model jointly estimates logit‐transformed sensitivity and specificity and accounts for a possible correlation between them.^22^ Substantial heterogeneity was anticipated, and random‐effects models were applied for pooled estimates. Forest plots with 95% confidence interval (CI) were generated. Subgroup analyses were based on classification systems and the PTEN assessment method. A summary receiver operating characteristic (SROC) curve was created, and the area under the curve (AUC) was calculated. A two‐tailed *P*‐value <0.05 was considered statistically significant. Heterogeneity among the outcomes of the studies included in this meta‐analysis was evaluated using the *Q*‐test and the *I* ^2^‐statistic. Significant heterogeneity was indicated by *Q*‐tests, *P* < 0.05 and *I* ^2^ >50%. Analyses were performed using R version 4.4.1 software with the packages “lme4,” “metafor,” and “mada” 1.^23^

Publication bias was assessed using Deeks' funnel‐plot asymmetry test based on the DOR and effective sample size.^24^

## 3 RESULTS

### 3.1 Study selection

**FIGURE 1:**
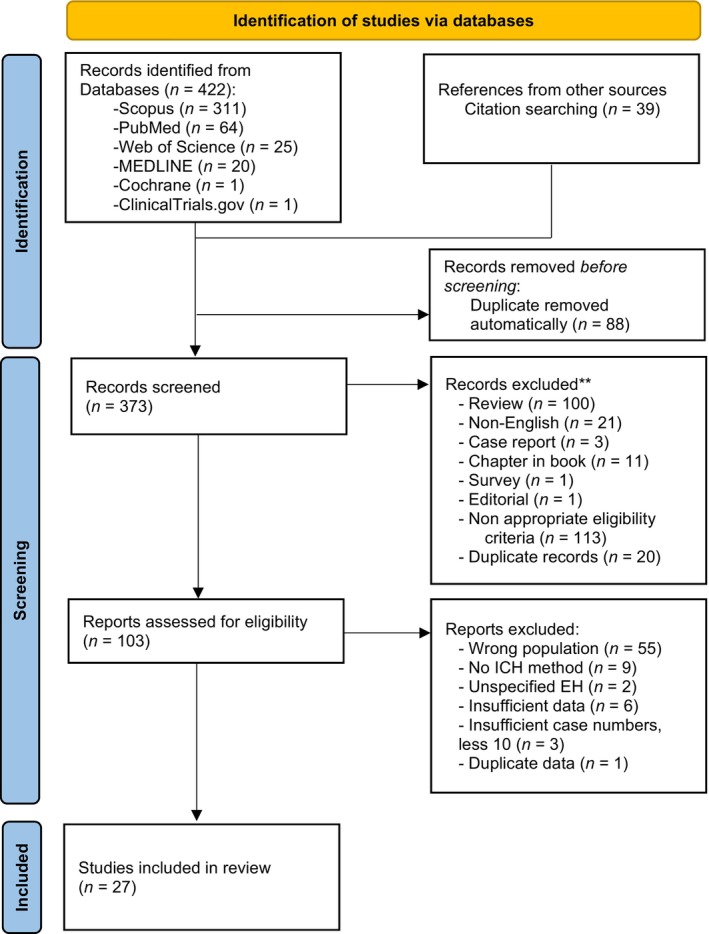
Flow diagram of the studies identified in the systematic review and article selection process. *Consider, if feasible to do so, reporting the number of records identified from each database or register searched (rather than the total number across all databases/registers). **If automation tools were used, indicate how many records were excluded by a human and how many were excluded by automation tools. *From*: Page et al.^25^

The search identified 422 relevant articles from databases and 39 through additional sources, with a total 461 references. After removing 88 records as duplicates, 373 records were screened by titles and abstracts. Of these, 103 publications were retained for full‐text review, 76 were excluded based on the selection criteria. Twenty‐seven articles were included in the meta‐analysis. The study selection process is shown in Figure 1.

### 3.2 Study characteristics

Twenty‐seven articles involving 2274 patients were analyzed. Nineteen studies were retrospective, three were prospective, one had a mixed retrospective‐prospective design, and the study design was not reported in four studies. Studies were published between 2000 and 2023 and conducted across Asia, Europe, North America, and the Middle East. The characteristics of the included studies are described in Table 1 and Table S2.

**TABLE 1:** Characteristics of studies included in the meta‐analysis.

First author, year	Country	Design	*N* cases	AH/EIN	AH/EIN	NAEH	NAEH	Method of assessment PTEN IHC staining	Definition of PTEN loss	PTEN staining interpretation
				PTEN loss	Total	PTEN loss	Total			
*WHO 1994 Classification*										
Abd El‐Masqoud, 2009^26^	Egypt	NR	20	2	8	0	12	Percentage and intensity, score 0–300	Complete loss	Loss/presenсe
Khan, 2021^27^	India	NR	268	5	49	0	219	Percentage and intensity	Absent staining	Intensity scale
Huang M., 2013^28^	USA	R	24	12	22	0	2	Percentage	<10% pos	Loss/presenсe
Kapucuoglu, 2007^29^	Turkey	R	37	2	10	0	27	Percentage and intensity, score 0–300	Complete loss	Loss/presenсe
Lacey, 2008^30^	USA	R	308	41	73	105	235	Null gland	Any null gland	Loss/presenсe
Lee, 2012^31^	Korea	R	42	15	21	5	21	Percentage	>5% absent staining in glands	Graded scale
Pavlakis, 2010^32^	Greece	R	83	38	58	15	25	Percentage	>80% absent staining in glands	Loss/presenсe
Pieczyńska, 2011^33^	Poland	R	132	1	16	4	116	Percentage and intensity, score 0–300	Complete loss	Loss/presenсe
Rani, 2019^34^	India	R	47	9	9	4	38	Percentage and intensity	<10% pos, intensity 0	Graded scale
Rao, 2011^35^	India	R‐P	76	13	13	46	63	Null gland	Any null gland	Graded scale
Sarmadi, 2009^36^	Iran	R	29	2	8	0	21	Percentage and intensity	<10% pos, intensity 0	Graded scale
Shawana, 2016^37^	Pakistan	R	26	4	6	1	20	Percentage and intensity	<10% pos, intensity 0	Graded scale
Stoenescu, 2017^38^	Romania	P	96	42	42	54	54	Intensity scale	Absent staining	Intensity scale
Tantbirojn, 2008^39^	Thailand	R	45	12	20	6	25	Intensity scale	Absent staining	Intensity scale
Upson, 2012^40^	USA	R	112	32	40	39	72	Percentage	>25% absent staining	Loss/presenсe
Total WHO 1994			1345	230	395	279	950			
*EIN Classification*										
Baak, 2005^41^	Norway	R	103	14	21	29	82	Null gland	Any null gland	Loss/presenсe
Mutter, 2000^42^	USA	R	19	9	12	2	7	Intensity scale	Absent staining	Intensity scale
Mutter, 2001^43^	USA	NR	76	22	35	23	41	Null gland	Any null gland	Loss/presenсe
Norimatsu, 2007^44^	Japan	R	70	13	38	4	32	Null gland	Any null gland	Loss/presenсe
Orbo, 2003^45^	Norway	R	68	11	39	3	29	Percentage	<10% pos	Loss/presenсe
Xiong, 2010^46^	China	R	83	9	24	17	59	Percentage	Complete loss	Graded scale
Total EIN			419	78	169	78	250			
*WHO 2014 Classification*										
Aguilar, 2023^47^	USA	R	195	56	111	13	84	Percentage	>10% absent staining in glands	Loss/presenсe
Allithy, 2022^48^	Egypt	P	54	0	29	0	25	Percentage and intensity, score 0–12	Score 0 (negative)	Graded scale
Huang Q., 2020^49^	China	NR	70	25	40	0	30	Percentage	≤10% pos	Loss/presenсe
Ramzy, 2022^50^	Egypt	R	50	8	8	14	42	Percentage	≤50% pos	Loss/presenсe
Sanderson, 2022^51^	UK	R	104	34	51	27	53	Null gland	Any null gland	Graded scale
Yadav, 2022^52^	India	P	37	8	10	19	27	Percentage and intensity, score 0–16	Score 0–3	Loss/presenсe
Total WHO 2014			510	131	249	73	261			
Total EH			2274	439	813	430	1461			

Abbreviations: AH/EIN, atypical hyperplasia/endometrial intraepithelial neoplasia; NAEH, non‐atypical endometrial hyperplasia; WHO 1994/WHO 2014, World Health Organization histological classification of endometrial hyperplasia (1994 or 2014 versions); Design: R—retrospective, P—prospective, R‐P—mixed retrospective‐prospective, NR—not reported; PTEN, phosphatase and tensin homolog; Method of assessment PTEN: Percentage, percentage of stained cells; Intensity, intensity of staining; Null gland, presence of PTEN‐null glands; Percentage and intensity, semiquantitative systems considering both parameters; Definition of PTEN loss: pos, positive staining.

Fifteen studies classified EH according to the WHO 1994 classification,^26^, ^27^, ^28^, ^29^, ^30^, ^31^, ^32^, ^33^, ^34^, ^35^, ^36^, ^37^, ^38^, ^39^, ^40^ six according to the EIN system,^41^, ^42^, ^43^, ^44^, ^45^, ^46^ and six according to WHO 2014.^47^, ^48^, ^49^, ^50^, ^51^, ^52^ These studies included 813 (35.75%) AH/EIN and 1461 (64.25%) non‐atypical EH. PTEN loss was found in 439 (54.00%) of precancerous lesions compared to 430 (29.43%) of benign EH.

In 12 studies, a single method was used to obtain samples: curettage in eight, biopsy in three, and surgical resection in one study. Multiple sampling methods were reported in nine studies: four included both curettage and biopsy, and five reported using biopsy and/or curettage along with hysterectomy. Six studies did not specify the sampling method.

Observer variability for PTEN interpretation was reported in 12 studies: two studies reported consensus among three pathologists, five involved two observers, and five were evaluated by a single pathologist. Fifteen studies did not report the number of observers.

PTEN immunoreactivity was assessed semiquantitatively by percentage of stained cells and staining intensity in nine studies. In nine studies, PTEN expression was evaluated based on the percentage of stained cells. Six studies assessed PTEN based on the presence of PTEN‐null glands. Three studies evaluated PTEN based on staining intensity. Finally, PTEN expression was dichotomized as present or absent in 15 studies and reported on a graded scale in 12 studies.

### 3.3 Risk of bias of included studies

The most frequent concerns regarding risk of bias arose in the participant selection domain as a result of using a case–control design in five studies^28^, ^30^, ^40^, ^49^, ^52^ and due to inappropriate exclusion in two studies,^31^, ^37^ with a total of seven (25.93%) studies classified as high or unclear risk. Eleven studies (40.74%) were deemed to be at low risk in all four bias domains of QUADAS‐2. The outcomes of bias assessment using the QUADAS‐2 tool are shown in Table 2. Deeks' regression test showed a *P*‐value of 0.2715, indicating no evidence of publication bias (Figure S1).

**TABLE 2:** Risk of bias and applicability concerns summary: Review authors “judgments” about each domain for each included study.

Study	Risk of bias				Applicability		
	Patient selection	Index test	Reference standard	Flow & timing	Patient selection	Index test	Reference standard
Abd El‐Masqoud, 2009^26^	Low	Low	Unclear	Low	Low	Low	Low
Khan, 2021^27^	Low	Unclear	Low	Low	Low	Low	Low
Huang M., 2013^28^	High	Low	Low	Low	Unclear	Low	Low
Kapucuoglu, 2007^29^	Low	Low	Unclear	Low	Low	Low	Low
Lacey, 2008^30^	Unclear	Low	Low	Low	Unclear	Low	Low
Lee, 2012^31^	Unclear	Low	Low	Low	Unclear	Low	Low
Pavlakis, 2010^32^	Low	Low	Low	Low	Unclear	Low	Low
Pieczyńska, 2011^33^	Low	Low	Low	Low	Low	Low	Low
Rani, 2019^34^	Low	Low	Unclear	Low	Low	Low	Low
Rao, 2011^35^	Low	Low	Unclear	Low	Low	Low	Low
Sarmadi, 2009^36^	Low	Low	Low	Low	Low	Low	Low
Shawana, 2016^37^	Unclear	Low	Low	Low	Unclear	Low	Low
Stoenescu, 2017^38^	Low	Unclear	Unclear	Low	Low	Low	Low
Tantbirojn, 2008^39^	Low	Unclear	Unclear	Low	Low	Unclear	Unclear
Upson, 2012^40^	Unclear	Low	Low	Low	Low	Low	Low
Baak, 2005^41^	Low	Low	Low	Low	Low	Low	Low
Mutter, 2000^42^	Low	Unclear	Low	Low	Low	Low	Low
Mutter, 2001^43^	Low	Low	Low	Low	Low	Low	Low
Norimatsu, 2007^44^	Low	Low	Low	Low	Low	Low	Low
Orbo, 2003^45^	Low	Low	Low	Low	Low	Low	Low
Xiong, 2010^46^	Low	Low	Low	Low	Low	Low	Low
Aguilar, 2023^47^	Low	Low	Low	Low	Low	Low	Low
Allithy, 2022^48^	Low	Low	Low	Low	Low	Low	Low
Huang Q., 2020^49^	Unclear	Low	Low	Low	Low	Low	Low
Ramzy, 2022^50^	Low	Low	Low	Low	Low	Low	Low
Sanderson, 2022^51^	Low	Low	Low	Low	Low	Low	Low
Yadav, 2022^52^	Unclear	Low	Low	Low	Low	Low	Low

### 3.4 Synthesis of results

#### 3.4.1 Diagnostic accuracy results

**FIGURE 2:**
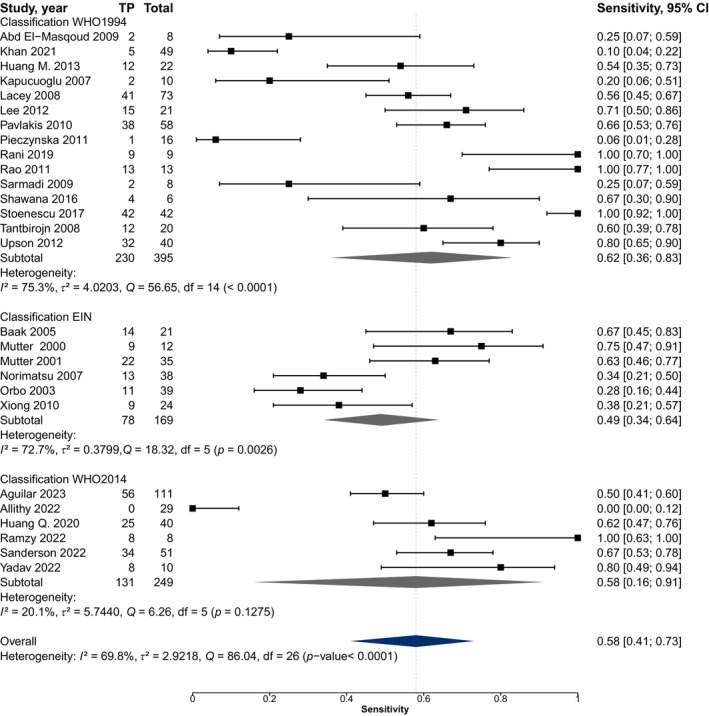
Forest plot for pooled sensitivity of PTEN loss by immunohistochemical assessment across three classification systems for differentiating AH/EIN from non‐atypical endometrial hyperplasia. AH/EIN, atypical hyperplasia/endometrioid intraepithelial neoplasia; CI, confidence interval; df, degrees of freedom; *I* ^2^, heterogeneity; *Q*, Cochran's heterogeneity statistic; tau^2^, tau‐squared; total, total number of AH/EIN; TP, true positives.

**FIGURE 3:**
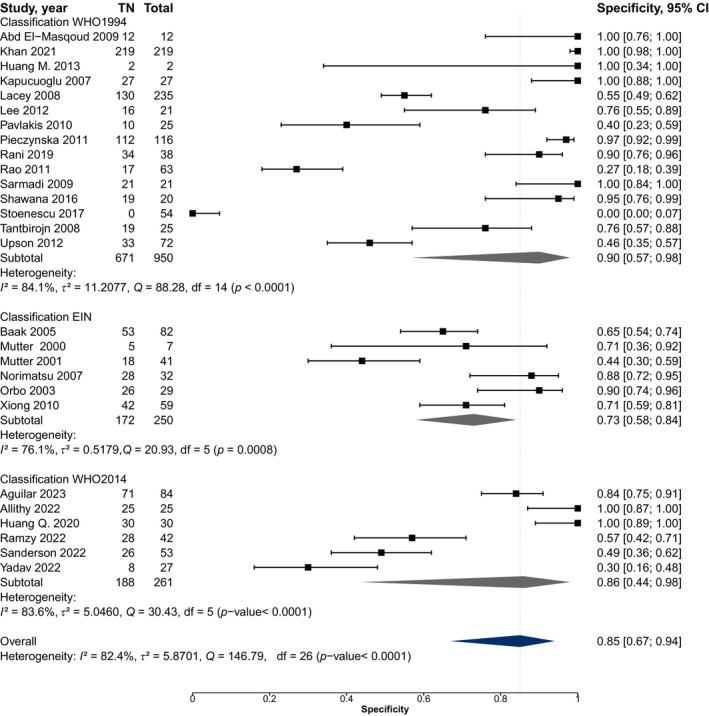
Forest plot for pooled specificity of PTEN loss by immunohistochemical assessment across three classification systems for differentiating AH/EIN from non‐atypical endometrial hyperplasia. AH/EIN, atypical hyperplasia/endometrioid intraepithelial neoplasia; CI, confidence interval; df, degrees of freedom; *I* ^2^, heterogeneity; *Q*, Cochran's heterogeneity statistic; tau^2^, tau‐squared; total, total number of AH/EIN; TP, true positives.

The pooled sensitivity and specificity of PTEN loss for detecting endometrial precancer were 58.0% (95% CI, 40.8–73.4) and 84.7% (95% CI, 67.0–93.8), respectively (Figures 2 and 3). The positive likelihood ratio, the negative likelihood ratio, the diagnostic odds ratio, and their corresponding 95% CIs were 1.926 (95% CI, 1.510–2.810), 0.710 (95% CI, 0.583–0.732), and 3.695 (95% CI, 2.501–5.460).

Significant heterogeneity was found for sensitivity (*I* ^2^ = 69.8%), specificity (*I* ^2^ = 82.4%) PLR (*I* ^2^ = 87.3%), and NLR (*I* ^2^ = 72.2%). The area under the SROC curve (AUC) 0.679 (Figure S2).

#### 3.4.2 Subgroup analysis

Subgroup analysis was conducted based on the classification systems used for EH and the method of PTEN assessment (Table S3).

Fifteen studies evaluated 1.45 EH using the WHO 1994 classification, including 395 (29.37%) AH/EIN and 950 (70.62%) EH without atypia. The pooled sensitivity and specificity were 62.4% (95% CI, 36.2%–82.9%) and 90.4% (95% CI, 57.3%–98.5%), respectively (Figures 2 and 3). The pooled DOR was 4.92 (95% CI, 2.28–9.32). The SROC curve showed an AUC of 0.72 (Figure S3).

Six studies assessed 419 EH using the EIN system; 169 (40.33%) were premalignant and 250 (59.62%) were benign. The pooled sensitivity and specificity of PTEN loss were 48.9% (95% CI, 34.5%–63.5%) and 72.9% (95% CI, 57.7%–84.2%), respectively (Figures 2 and 3). Pooled DOR was 2.4 (1.41–3.83). The area under the SROC curve (AUC) was 0.586 (Figure S4).

Six studies assessed 510 EH using the WHO 2014 system, including 249 (48.82%) AH/EIN and 261 (51.18%) non‐atypical EH. Forest plots revealed pooled sensitivity and specificity of 57.8% (95% CI, 15.9%–90.8%) and 85.8% (95% CI, 43.8%–97.9%), respectively (Figures 2 and 3). Pooled DOR was 4.51 (1.87–9.20). The SROC curve analysis demonstrated an AUC of 0.701 (Figure S5).

In addition, we conducted subgroup analysis stratified by the method of PTEN IHC assessment (Table S3). Nine studies provided data on the diagnostic values of PTEN using percentage and intensity of staining. The pooled sensitivity, specificity, and DOR were 28.2% (95% CI, 7.8–64.8), 99.2% (95% CI, 82.9–100), and 9.71 (95% CI, 3.26–22.70), respectively, with AUC of 0.744.

Based on nine studies that assessed PTEN using the percentage of positively stained cells, the pooled sensitivity was 60.3% (95% CI, 46.8–72.4), specificity was 75.6% (95% CI, 57.9–87.5), the DOR was 2.77 (95% CI, 1.91–3.90), and the AUC was 0.657.

In six studies applying the presence of PTEN null glands as the diagnostic criterion, the pooled sensitivity was 64.1% (95% CI, 47.0–78.2) and specificity was 55.0% (95% CI, 38.8–70.3), with a DOR of 1.89 (95% CI, 1.24–2.77) and an AUC of 0.576.

Based on three studies that assessed PTEN using staining intensity, the pooled sensitivity was 90.0% (95% CI, 38.9–99.2) and specificity was 20.3% (95% CI, 0.3–95.8), with a DOR of 4.01 (95% CI, 0.64–13.6) and an AUC of 0.694.

#### 3.4.3 Combinations of markers

Three studies^44^, ^47^, ^51^ reported the diagnostic performance of PTEN loss in combination with other biomarkers, including PAX2, β‐catenin, and HAND2, for distinguishing AH/EIN from non‐atypical EH (Table S4). However, data on the benefits of combinations are limited, and no pooled analysis can be performed.

## 4 DISCUSSION

This systematic review and meta‐analysis indicates that PTEN loss is more frequently observed in histological precancerous lesions than in benign EH. PTEN loss demonstrated good specificity (84.7%) but moderate sensitivity (58%) for detecting AH/EIN, with an AUC of 0.679. Importantly, diagnostic performance varied substantially across assessment approaches; for example, the method based only on staining intensity demonstrated low specificity (20%).

Among PTEN assessment methods, the combined evaluation of staining intensity and the percentage of positively stained cells showed acceptable diagnostic performance, although sensitivity remained limited.

The pooled specificity of 84.7% suggests that assessing PTEN loss can help reduce false‐positive diagnoses in morphologically ambiguous cases and guide a diagnosis toward non‐atypical EH. Difficulties in morphological interpretation might result in incorrect diagnoses of atypia in borderline cases,^9^ potentially leading to unnecessary hysterectomies. Kimura et al. reported that 45.6% of patients who underwent hysterectomy following a diagnosis of AH/EIN were found to have non‐atypical EH or normal endometrium on final pathology.^53^ Therefore, PTEN assessment might serve as a tool to support clinical decision‐making and prevent overtreatment, particularly for patients seeking fertility preservation or those with high surgical risk. However, the low sensitivity necessitates careful follow‐up, as the risk of undiagnosed atypia progressing to cancer remains.

### 4.1 Сomparison with existing literature

We observed PTEN loss in 54% of precancerous lesions compared to 29.43% of benign EH. These results are consistent with earlier reports, in which PTEN loss was found in 27% of non‐atypical EH, 42%–51% of AH/EIN, and 60%–80% of EC.^47^, ^54^, ^55^, ^56^, ^57^ Therefore, these frequencies support its role as an early event in endometrial carcinogenesis. According to The Cancer Genome Atlas (TCGA), PTEN mutations are present in 11%–94% of endometrioid EC, depending on molecular subtype.^11^ This variability likely reflects underlying molecular diversity in the pathogenesis of endometrial neoplasia, with some lesions progressing via PTEN‐independent pathways, resulting in false‐negative outcomes and reduced sensitivity of PTEN testing.

Our findings are in agreement with the systematic review by Raffone et al., which also reported low sensitivity for PTEN loss as a diagnostic marker.^58^ However, their reported specificity (66%) was lower than in our meta‐analysis (84.7%), likely reflecting differences in study inclusion. Our analysis incorporated studies applying the updated WHO 2014 classification of EH and included nine additional recent publications, resulting in a more comprehensive and contemporary synthesis.

Differences in histological classifications applied (WHO 1994, EIN, WHO 2014) might affect diagnostic performance. Subgroup analysis demonstrated the poorest diagnostic performance under EIN classification. The EIN system relies on a combination of morphometric, architectural, and molecular criteria, resulting in more narrowly defined lesions, whereas WHO 1994 and WHO 2014 apply broader morphologic thresholds. Although WHO 1994 and WHO 2014 systems showed comparable sensitivity, specificity was slightly lower with WHO 2014. The consolidation of four categories in WHO 1994 into two categories (atypical and non‐atypical) in WHO 2014 might lead to ambiguity in borderline cases, increasing the risk of false‐positive atypia diagnoses, particularly in the presence of metaplastic or inflammatory changes. Further, morphological heterogeneity within AH/EIN itself might contribute to diagnostic uncertainty (e.g., variations in the grade of nuclear atypia, which are not reflected in current classification systems). D'Angelo et al. found that 61% of patients with preoperative high‐grade AH/EIN were diagnosed with grade 1 EC at hysterectomy.^59^

Further complexity arises in morphologically ambiguous biopsies. Wyvekens et al. examined subdiagnostic specimens characterized by focal areas of gland crowding and found that PTEN loss occurred at similar rates, in 30% of cases with subsequent AH/EIN or EC and in 32% of cases with benign outcomes.^60^ Regular repeat sampling remains a valuable approach in this setting. Another potential source of the observed heterogeneity is the variability in PTEN assessment methodology and the definition of PTEN loss across studies. IHC evaluation approaches differed considerably, including assessment based on staining intensity alone, percentage of positive or negative cells, combined evaluation of percentage and intensity, and identification of PTEN‐null glands. Definitions of PTEN loss also varied, ranging from complete absence of staining to thresholds such as <10% or <50% positive cells, as well as different interpretative frameworks (loss/presence vs graded scales).

In our subgroup analysis, PTEN assessment based on the combined evaluation of the percentage of stained cells and staining intensity demonstrated comparatively better diagnostic performance among the evaluated methods but low sensitivity. However, even within this method, IHC evaluation varied: some studies applied integrated scoring systems (*H*‐score, immunoreactivity scoring systems), whereas others assessed percentage and intensity without formal scoring formulas. Definitions of PTEN loss within this group also varied, including complete absence of staining or threshold such as <10% positive staining. Other assessment methods showed lower diagnostic performance, with the poorest accuracy observed in studies relying on identifying PTEN‐null glands. Meta‐analysis consistently showed that using any null glands as the criterion for PTEN loss yielded the lowest diagnostic accuracy for detecting precancerous EH, possibly because such glands can appear histologically benign and might regress spontaneously.^61^

### 4.2 Strengths and limitations

To our knowledge, this is the first study to evaluate the diagnostic performance of PTEN loss in distinguishing AH/EIN from benign EH, including studies classified under the updated WHO 2014/WHO 2020 system. A further strength is the comprehensive subgroup analysis of different PTEN IHC assessment methods. This study also includes the largest number of studies to date and applies a robust statistical methodology.

Limitations include substantial heterogeneity arising from differing histological classifications, IHC protocols and PTEN‐loss definitions. Given the significant heterogeneity across studies, a random‐effects model was used to account for between‐study heterogeneity; however, the findings should be interpreted with caution. Although publication bias was not indicated, in the presence of heterogeneity, tests of funnel‐plot asymmetry have low power, and the results should, therefore, be interpreted accordingly.

Many studies used multiple sampling methods, which further complicates the comparison. PTEN loss might be highly focal, and sampling via biopsy, curettage, or hysterectomy can yield variable tissue representation, affecting diagnostic accuracy. While Pipelle biopsy yields good‐quality histological specimens, small or localized focal lesions might remain undetected.^62^

Interobserver variability in PTEN IHC interpretation is another concern. While a few studies used consensus scoring or multiple observers, many did not specify the number or the training of evaluators. Further, variability in antibodies used for IHC, including clone 28H6, previously associated with non‐specific staining, might contribute to study heterogeneity.^63^

Although PTEN status appears to have potential utility in guiding fertility‐sparing treatment decisions, particularly in women of reproductive age, most of the included studies did not stratify patients by age. Additionally, most included studies did not provide detailed demographic data such as body mass index, race, or ethnicity, which might further limit the generalizability of our findings across diverse populations. Finally, the inclusion of only English‐language studies might have introduced bias.

### 4.3 Implications for clinical practice and further research

PTEN loss shows potential as a complementary diagnostic tool for distinguishing atypical from non‐atypical EH, particularly in borderline cases where it might help reduce the risk of overdiagnosis of atypia. In such cases, a retained PTEN immunoreactivity might support a short‐interval follow‐up or a repeat sampling rather than an immediate surgery, especially when fertility preservation or surgical comorbidity is a clinical concern. PTEN loss raises a suspicion for AH/EIN; however, due to its limited sensitivity, it should not serve as the sole determinant for definitive clinical management.

Based on the current study, the combined evaluation of the percentage of stained cells and staining intensity is the most promising PTEN IHC method for differentiating AH/EIN from benign EH. However, further research is required to standardize the protocol and establish clinically meaningful thresholds. Importantly, data specific to reproductive‐age women remain limited. Future studies should stratify by age, comorbidities, and sampling methods to better define the clinical utility of PTEN loss across different patient populations and clinical settings.

Given suboptimal sensitivity PTEN loss, combining PTEN with other biomarkers might improve diagnostic precision. For instance, 31%–35.2% of AH/EIN lesions were negative for both PTEN and PAX2, and 54%–88.9% were null for either marker.^17^, ^55^ Norimatsu et al. reported that a combination of PTEN loss with β‐catenin positive staining was identified in 57.9% of AH/EIN lesions, while this pattern was present in only 12.5% of benign EH.^44^ A panel incorporating PTEN, PAX2, and HAND2 identified 86.3% of AH/EIN and 87% of EH without atypia.^49^ Aguilar et al. found that at least one of three markers (PTEN, PAX2, or β‐catenin) was aberrant in 92.8% of AH/EIN cases and in 47.5 non‐atypical EH.^64^ These findings suggest that the development of multi‐marker models could improve diagnostic accuracy.

## 5 CONCLUSION

This meta‐analysis demonstrates that PTEN loss assessment might serve as a complementary tool for differentiating AH/EIN and benign conditions in ambiguous cases. Its good specificity supports its role in confirming benign diagnoses and reducing overtreatment, particularly in women seeking fertility preservation or patients with significant surgical risk. However, its limited sensitivity precludes its use as a standalone diagnostic marker. Combining evaluation of staining percentage and intensity yielded the best diagnostic performance. Further research is needed to standardize PTEN assessment protocols and to explore multi‐biomarker combinations to optimize the clinical utility.

## AUTHOR CONTRIBUTIONS

K.S., M.T.R., and S.B. were involved in the conception and planning of the study. K.G. provided expert methodological support for the systematic review and meta‐analysis. K.S. and M.T.R. undertook the search, screen, selection, and data extraction and assessments of the included studies. T.K. provided support for analysis. K.S. wrote the initial manuscript draft, S.B., T.K., K.G., P.W., S.B. and M.T.R. reviewed and edited the paper. All authors edited and accepted the manuscript prior to submission.

## FUNDING INFORMATION

KS was supported by DAAD (German Academic Exchange Service) research grant (ID: 57693450). The funders have no involvement in any stage of this systematic review.

## CONFLICT OF INTEREST STATEMENT

The authors have no conflicts of interest to declare.

## ETHICS STATEMENT

The authors have nothing to report.

## Supporting information


Data S1.

## Data Availability

Data used in this review can be made available by the author upon reasonable request.

## References

[1] Bray F , Laversanne M , Sung H , et al. Global cancer statistics 2022: GLOBOCAN estimates of incidence and mortality worldwide for 36 cancers in 185 countries. CA Cancer J Clin. 2024;74(3):229‐263. 38572751 10.3322/caac.21834

[2] Lacey JV , Sherman ME , Rush BB , et al. Absolute risk of endometrial carcinoma during 20‐year follow‐up among women with endometrial hyperplasia. JCO. 2010;28(5):788‐792. 10.1200/JCO.2009.24.1315 PMC2834395 20065186

[3] Nees LK , Heublein S , Steinmacher S , et al. Endometrial hyperplasia as a risk factor of endometrial cancer. Arch Gynecol Obstet. 2022;306(2):407‐421. 35001185 10.1007/s00404-021-06380-5 PMC9349105

[4] Concin N , Matias‐Guiu X , Vergote I , et al. ESGO/ESTRO/ESP guidelines for the management of patients with endometrial carcinoma. Int J Gynecol Cancer. 2021;31(1):12‐39. 33397713 10.1136/ijgc-2020-002230

[5] De Rocco S , Buca D , Oronzii L , et al. Reproductive and pregnancy outcomes of fertility‐sparing treatments for early‐stage endometrial cancer or atypical hyperplasia: a systematic review and meta‐analysis. Eur J Obstet Gynecol Reprod Biol. 2022;273:90‐97. 35526471 10.1016/j.ejogrb.2022.04.019

[6] Catena U , Macklon KLT , Rodolakis A , Scambia G . A practical guideline on the fertility‐sparing treatment of patients with endometrial carcinoma and atypical endometrial hyperplasia. Int J Gynaecol Obstet. 2025;169:453‐455. doi:10.1002/ijgo.70059 40071490

[7] Mutter GL . Endometrial intraepithelial neoplasia (EIN): will it bring order to chaos? Gynecol Oncol. 2000;76(3):287‐290. 10684697 10.1006/gyno.1999.5580

[8] Kurman R , Carcangiu M , Herrington C , Young R . World Health Organisation classification of tumors of female reproductive organs. 4th ed. International Agency for Research on Cancer (IARC) Press; 2014.

[9] McCoy CA , Coleman HG , McShane CM , et al. Factors associated with interobserver variation amongst pathologists in the diagnosis of endometrial hyperplasia: a systematic review. PLoS One. 2024;19(4):e0302252. 38683770 10.1371/journal.pone.0302252 PMC11057740

[10] Mutter GL , Lax SF . Endometrial atypical hyperplasia/endometrioid intraepithelial neoplasia. In: WHO Classification of Tumours Editorial Board FGT , ed. WHO classification of tumours series. Vol 4. 5th ed. International Agency for Research on Cancer; 2020:250‐251.

[11] Levine DA . Integrated genomic characterization of endometrial carcinoma. Nature. 2013;497(7447):67‐73. 23636398 10.1038/nature12113 PMC3704730

[12] Russo M , Newell JM , Budurlean L , et al. Mutational profile of endometrial hyperplasia and risk of progression to endometrioid adenocarcinoma. Cancer. 2020;126(12):2775‐2783. 32187665 10.1002/cncr.32822

[13] Song MS , Salmena L , Pandolfi PP . The functions and regulation of the PTEN tumour suppressor. Nat Rev Mol Cell Biol. 2012;13(5):283‐296. 22473468 10.1038/nrm3330

[14] Lupini L , Scutiero G , Iannone P , et al. Molecular biomarkers predicting early development of endometrial carcinoma: a pilot study. Eur J Cancer Care. 2019;28(6):e13137. 10.1111/ecc.13137 31412428

[15] Gotoh O , Sugiyama Y , Tonooka A , et al. Genetic and epigenetic alterations in precursor lesions of endometrial endometrioid carcinoma. J Pathol. 2024;263(3):275‐287. 38734880 10.1002/path.6278

[16] Ayhan A , Mao T , Suryo Rahmanto Y , et al. Increased proliferation in atypical hyperplasia/endometrioid intraepithelial neoplasia of the endometrium with concurrent inactivation of ARID1A and PTEN tumour suppressors. J Pathol CR. 2015;1(3):186‐193. 10.1002/cjp2.22 PMC4939882 27499903

[17] Chen H , Lucas E , Strickland AL , et al. Specific biomarker expression patterns in the diagnosis of residual and recurrent endometrial precancers after progestin treatment: a longitudinal study. Am J Surg Pathol. 2020;44(10):1429‐1439. 32931681 10.1097/PAS.0000000000001537

[18] McInnes MDF , Moher D , Thombs BD , et al. Preferred reporting items for a systematic review and meta‐analysis of diagnostic test accuracy studies: the PRISMA‐DTA Statement. JAMA. 2018;319(4):388. 29362800 10.1001/jama.2017.19163

[19] Mutter GL , Zaino RJ , Baak JP , Bentley RC , Robboy SJ . Benign endometrial hyperplasia sequence and endometrial intraepithelial neoplasia. Int J Gynecol Pathol. 2007;26(2):103‐114. doi:10.1097/01.pgp.0000236657.85961.23 17413975

[20] Chen H , Strickland AL , Castrillon DH . Histopathologic diagnosis of endometrial precancers: Updates and future directions. Semin Diagn Pathol. 2022;39(3):137‐147. 34920905 10.1053/j.semdp.2021.12.001 PMC9035046

[21] Whiting PF . QUADAS‐2: a revised tool for the quality assessment of diagnostic accuracy studies. Ann Intern Med. 2011;155(8):529. 22007046 10.7326/0003-4819-155-8-201110180-00009

[22] Schlattmann P . Tutorial: statistical methods for the meta‐analysis of diagnostic test accuracy studies. Clin Chem Lab Med (CCLM). 2023;61(5):777‐794. 36656998 10.1515/cclm-2022-1256

[23] R Core Team . R: a language and environment for statistical computing. R Foundation for Statistical Computing; 2025. https://www.R‐project.org/

[24] Deeks JJ , Macaskill P , Irwig L . The performance of tests of publication bias and other sample size effects in systematic reviews of diagnostic test accuracy was assessed. J Clin Epidemiol. 2005;58(9):882‐893. 16085191 10.1016/j.jclinepi.2005.01.016

[25] Page MJ , McKenzie JE , Bossuyt PM , et al. The PRISMA 2020 statement: an updated guideline for reporting systematic reviews. BMJ. 2021;372:n71. doi:10.1136/bmj.n71 33782057 PMC8005924

[26] Abd El‐Maqsoud NMR , El‐Gelany S . Differential expression patterns of PTEN in cyclic, hyperplastic and malignant endometrium: its relation with ER, PR and clinicopathological parameters. J Egypt Natl Canc Inst. 2009;21(4):323‐331. 21415869

[27] Abu Shahma Khan AS , Alam K , Akhtar K , Khan T , Alam F . Diagnostic implication of phosphatase and tensin homolog expression in endometrial lesions. Int J Curr Res Rev. 2021;13(6):124‐128.

[28] Huang M , Djordjevic B , Yates MS , et al. Molecular pathogenesis of endometrial cancers in patients with lynch syndrome. Cancer. 2013;119(16):3027‐3033. 23760948 10.1002/cncr.28152 PMC4120439

[29] Kapucuoglu N , Aktepe F , Kaya H , Bircan S , Karahan N , Çiriş M . Immunohistochemical expression of PTEN in normal, hyperplastic and malignant endometrium and its correlation with hormone receptors, bcl‐2, bax, and apoptotic index. Pathol Res Pract. 2007;203(3):153‐162. 17317031 10.1016/j.prp.2007.01.003

[30] Lacey JV , Mutter GL , Ronnett BM , et al. PTEN expression in endometrial biopsies as a marker of progression to endometrial carcinoma. Cancer Res. 2008;68(14):6014‐6020. 18632658 10.1158/0008-5472.CAN-08-1154 PMC2493615

[31] Lee H , Choi HJ , Kang CS , Lee HJ , Lee WS , Park CS . Expression of miRNAs and PTEN in endometrial specimens ranging from histologically normal to hyperplasia and endometrial adenocarcinoma. Mod Pathol. 2012;25(11):1508‐1515. 22766795 10.1038/modpathol.2012.111

[32] Pavlakis K , Messini I , Vrekoussis T , et al. PTEN‐loss and nuclear atypia of EIN in endometrial biopsies can predict the existence of a concurrent endometrial carcinoma. Gynecol Oncol. 2010;119(3):516‐519. 20833413 10.1016/j.ygyno.2010.08.023

[33] Pieczyńska B , Wojtylak S , Zawrocki A , Biernat W . Analysis of PTEN, estrogen receptor α and progesterone receptor expression in endometrial hyperplasia using tissue microarray. Pol J Pathol. 2011;62(3):133‐138. 22102068

[34] Rani E , Ahmad Mir S , Bhat S , Shera S . Study of expression of PTEN in endometrium AT a tertiary care CENTRE. IJAR. 2019;7(10):902‐906.

[35] Rao Anuradha CK , Arya G , Padma P . Immunohistochemical phospho tensin tumor suppressor gene staining patterns in endometrial hyperplasias: a 2‐year study. Indian J Pathol Microbiol. 2011;54(2):264. 21623071 10.4103/0377-4929.81588

[36] Sarmadi S , Izadi‐Mood N , Sotoudeh K , Tavangar S . Altered PTEN expression; a diagnostic marker for differentiating normal, hyperplastic and neoplastic endometrium. Diagn Pathol. 2009;4(1):41. 19930726 10.1186/1746-1596-4-41 PMC2789036

[37] Shawana S , Kehar SI , Shaikh F . Differential expression of phophatase and tensin homologue in normal, hyperplastic and neoplastic endometrium. J Pak Med Assoc. 2014;64(10):1103‐1108. 25823145

[38] Stoenescu VE , Niculescu M , Novac L , et al. Immunohistochemical reaction of the glandular epithelium in endometrial hyperplasia compared to endometrial carcinoma. Rom J Morphol Embryol. 2017;58(3):791‐800. 29250656

[39] Tantbirojn P , Triratanachat S , Trivijitsilp P , Niruthisard S . Detection of PTEN immunoreactivity in endometrial hyperplasia and adenocarcinoma. J Med Assoc Thai. 2008;91(8):1161‐1165. 18788685

[40] Upson K , Allison KH , Reed SD , et al. Biomarkers of progestin therapy resistance and endometrial hyperplasia progression. Am J Obstet Gynecol. 2012;207(1):36.e1‐36.e8. 10.1016/j.ajog.2012.05.012 PMC3398620 22727345

[41] Baak JPA , Van Diermen B , Steinbakk A , et al. Lack of PTEN expression in endometrial intraepithelial neoplasia is correlated with cancer progression. Hum Pathol. 2005;36(5):555‐561. 15948123 10.1016/j.humpath.2005.02.018

[42] Mutter GL . Altered PTEN expression as a diagnostic marker for the earliest endometrial precancers. J Natl Cancer Inst. 2000;92(11):924‐930. 10841828 10.1093/jnci/92.11.924

[43] Mutter GL , Ince TA , Baak JP , Kust GA , Zhou XP , Eng C . Molecular identification of latent precancers in histologically normal endometrium. Cancer Res. 2001;61(11):4311‐4314. 11389050

[44] Norimatsu Y , Moriya T , Kobayashi TK , et al. Immunohistochemical expression of PTEN and β‐catenin for endometrial intraepithelial neoplasia in Japanese women. Ann Diagn Pathol. 2007;11(2):103‐108. 17349568 10.1016/j.anndiagpath.2006.06.009

[45] Orbo A , Nilsen MN , Arnes MS , Pettersen I , Larsen K . Loss of expression of MLH1, MSH2, MSH6, and PTEN related to endometrial cancer in 68 patients with endometrial hyperplasia. Int J Gynecol Pathol. 2003;22(2):141‐148. 12649668 10.1097/00004347-200304000-00005

[46] Xiong Y , Xiong YY , Zhou YF . Expression and significance of beta‐catenin, Glut‐1 and PTEN in proliferative endometrium, endometrial intraepithelial neoplasia and endometrioid adenocarcinoma. Eur J Gynaecol Oncol. 2010;31(2):160‐164. 20527231

[47] Aguilar M , Chen H , Sahoo SS , et al. Β‐Catenin, Pax2, and PTEN panel identifies precancers among histologically subdiagnostic endometrial lesions. Am J Surg Pathol. 2023;47(5):618‐629. 36939046 10.1097/PAS.0000000000002034 PMC10101134

[48] Allithy AN , Ammar IMM , Mohammed MH . Diagnostic and prognostic values of PTEN expression in functional and pathological endometrial biopsies. Asian Pac J Cancer Biol. 2022;7(1):21‐27.

[49] Huang Q , He Y , Cao X , Yi C . PTEN and CD146 expression in endometrioid adenocarcinoma. EJGO. 2020;41(3):439.

[50] Ramzy NI , Ibrahiam WS , Ali HHM , Akle MMA , Khalifa SE . Possible role of PTEN expression in discriminating benign endometrial hyperplasia from atypical hyperplasia/endometrial intraepithelial neoplasiain a series of Egyptian patients. Curr Womens Health Rev. 2022;18(3):e031121195558.

[51] Sanderson PA , Esnal‐Zufiaurre A , Arends MJ , et al. Improving the diagnosis of endometrial hyperplasia using computerized analysis and immunohistochemical biomarkers. Front Reprod Health. 2022;4:896170. 36303676 10.3389/frph.2022.896170 PMC9580641

[52] Yadav S , Makker A , Agarwal P , Singh U , Nayak S , Goel MM . Phosphatase and tensin homolog immunohistochemical expression and promoter methylation status in endometrioid endometrial carcinoma and its precursor lesions. Cureus. 2022;14:e30778. 36447725 10.7759/cureus.30778 PMC9701162

[53] Kimura T , Kamiura S , Komoto T , et al. Clinical over‐ and under‐estimation in patients who underwent hysterectomy for atypical endometrial hyperplasia diagnosed by endometrial biopsy: the predictive value of clinical parameters and diagnostic imaging. Eur J Obstetr Gynecol Reprod Biol. 2003;108(2):213‐216. 10.1016/s0301-2115(02)00469-4 12781414

[54] Vierkoetter K , Wong J , Ahn HJ , Shimizu D , Kagami L , Terada K . Using gene expression in patients with endometrial intraepithelial neoplasia to assess the risk of cancer. Gynecol Oncol Rep. 2018;24:24‐26. 29845103 10.1016/j.gore.2018.02.006 PMC5966520

[55] Monte NM , Webster KA , Neuberg D , Dressler GR , Mutter GL . Joint loss of PAX2 and Pten expression in endometrial precancers and cancer. Cancer Res. 2010;70(15):6225‐6232. 20631067 10.1158/0008-5472.CAN-10-0149 PMC2912978

[56] Athanassiadou P , Athanassiades P , Grapsa D , et al. The prognostic value of PTEN, p53, and beta‐catenin in endometrial carcinoma: a prospective immunocytochemical study. Int J Gynecol Cancer. 2007;17(3):697‐704. 17504383 10.1111/j.1525-1438.2007.00845.x

[57] Peng S , Zheng Y , Liu J , et al. Molecular classification in fertility‐sparing treatment of early‐stage endometrial cancer: a potential tool for optimizing patient selection. Gynecol Oncol. 2024;191:240‐248. 39461269 10.1016/j.ygyno.2024.10.012

[58] Raffone A , Travaglino A , Saccone G , et al. Loss of PTEN expression as diagnostic marker of endometrial precancer: a systematic review and meta‐analysis. Acta Obstet Gynecol Scand. 2019;98(3):275‐286. 30511743 10.1111/aogs.13513

[59] D'Angelo E , Espinosa I , Cipriani V , Szafranska J , Barbareschi M , Prat J . Atypical endometrial hyperplasia, low‐grade: “much ADO about nothing”. Am J Surg Pathol. 2021;45(7):988‐996. 34105519 10.1097/PAS.0000000000001705

[60] Wyvekens N , Mutter GL , Nucci MR , Kolin DL , Parra‐Herran C . Lesions sub‐diagnostic of endometrioid intra‐epithelial neoplasia/atypical hyperplasia: value of morphology and immunohistochemistry in predicting neoplastic outcome. Histopathology. 2024;85(4):579‐589. doi:10.1111/his.15215 38785042

[61] Travaglino A , Raffone A , Saccone G , et al. PTEN immunohistochemistry in endometrial hyperplasia: which are the optimal criteria for the diagnosis of precancer? APMIS. 2019;127(4):161‐169. 30803040 10.1111/apm.12938

[62] Tahir MM , Bigrigg MA , Browning JJ , Brookes T , Smith PA . A randomised controlled trial comparing transvaginal ultrasound, outpatient hysteroscopy and endometrial biopsy with inpatient hysteroscopy and curettage. BJOG. 1999;106(12):1259‐1264. 10.1111/j.1471-0528.1999.tb08179.x 10609719

[63] Pallares J , Bussaglia E , Martínez‐Guitarte JL , et al. Immunohistochemical analysis of PTEN in endometrial carcinoma: a tissue microarray study with a comparison of four commercial antibodies in correlation with molecular abnormalities. Mod Pathol. 2005;18(5):719‐727. 15578076 10.1038/modpathol.3800347

[64] Aguilar M , Chen H , Rivera‐Colon G , et al. Reliable identification of endometrial precancers through combined Pax2, β‐catenin, and Pten immunohistochemistry. Am J Surg Pathol. 2022;46(3):404‐414. 34545858 10.1097/PAS.0000000000001810 PMC8860214

