# Supplementary material for: Diagnostic accuracy of phosphatase and tensin homolog loss in differentiating between atypical endometrial hyperplasia/endometrioid intraepithelial neoplasia and non‐atypical endometrial hyperplasia: A systematic review and meta‐analysis
Source: Int J Gynaecol Obstet. 2026 Feb 26;174(2):630–42. doi: 10.1002/ijgo.70901 (PMC13377286; doi:10.1002/ijgo.70901)
Supplement: Supplementary file 1 — Data S1. [file IJGO-174-630-s001.docx]

Table S1. Search strategy

| **Database Name** | **Search Strategy** |
| --- | --- |
| Pubmed | ((endometrial hyperplasia) OR (endometrial intraepithelial neoplasia) OR (EIN)) AND ((PTEN) OR (phosphatase and tensin homolog)) AND ((marker) OR (biomarker)) AND ((immunohistochemistry) OR (immunohistochemical)) AND (diagnosis) |
| Scopus | TITLE-ABS-KEY ( ( endometrial AND hyperplasia ) OR ( endometrial AND intraepithelial AND neoplasia ) OR ( ein ) ) AND ( ( pten ) OR ( phosphatase AND tensin AND homolog ) ) AND ( ( marker ) OR ( biomarker ) ) AND ( ( immunohistochemistry ) OR ( immunohistochemical ) ) AND ( diagnosis ) |
| Cochrane Library | ((endometrial hyperplasia) OR (endometrial intraepithelial neoplasia) OR (EIN)) AND ((PTEN) OR (phosphatase and tensin homolog)) AND ((marker) OR (biomarker)) AND ((immunohistochemistry) OR (immunohistochemical)) AND (diagnosis) |
| ClinicalTrial.gov | ((endometrial hyperplasia) OR (endometrial intraepithelial neoplasia) OR (EIN)) AND ((PTEN) OR (phosphatase and tensin homolog)) AND ((marker) OR (biomarker)) AND ((immunohistochemistry) OR (immunohistochemical)) AND (diagnosis) |
| Web of Sciences | (ALL = (endometrial hyperplasia) OR ALL = (endometrial intraepithelial neoplasia) OR ALL= (ein)) AND (ALL = (pten) OR ALL = (phosphatase AND tensin homolog)) AND (ALL= (marker) OR ALL= (biomarker)) AND (ALL= (immunohistochemistry) OR ALL= (immunohistochemical)) AND ALL= (diagnosis) |
| MEDLINE | ( TS = (endometrial hyperplasia) OR TS = (endometrial intraepithelial neoplasia) OR TS= (ein)) AND (TS = (pten) OR TS = (phosphatase AND tensin homolog)) AND (TS= (marker) OR TS= (biomarker)) AND (TS= (immunohistochemistry) OR TS= (immunohistochemical)) AND TS= (diagnosis) |

**Table S2:** Characteristics of studies included in the meta-analysis.

Abbreviations: AH/EIN, atypical hyperplasia / endometrial intraepithelial neoplasia; SH, simple hyperplasia; CH, complex hyperplasia; LS, Lynch syndrome; WHO1994/WHO2014, World Health Organization histological classification of endometrial hyperplasia (1994 or 2014 versions); Design, R — retrospective, P — prospective, R-P — mixed retrospective-prospective; NR, not reported; PTEN, phosphatase and tensin homolog;

Method of assessment PTEN: Percentage, percentage of stained cells; Intensity, intensity of staining; Null gland, presence of PTEN-null glands; Percentage and intensity, semiquantitative systems considering both parameters;

Definition of PTEN loss: pos, positive staining;

|  |  |  |  |  | AH/EIN | AH/EIN | NAEH | NAEH |  |  |  |  |  |  |
| --- | --- | --- | --- | --- | --- | --- | --- | --- | --- | --- | --- | --- | --- | --- |
| First author, Classification | Year | Country | Design | N cases | PTEN loss | Total | PTEN loss | Total | Methods sampling | Age (y) | Method of assessment PTEN IHC staining | Definition of PTEN loss | PTEN Staining Interpretation | N  Pathologists PTEN assessment |
| **WHO1994** |  |  |  |  |  |  |  |  |  |  |  |  |  |  |
| Abd El-Masqoud^25^ | 2009 | Egypt | NR | 20 | 2 | 8 | 0 | 12 | Biopsy, curettage | NR | Percentage and intensity, score 0-300 | Complete loss | Loss/ presenсe | NR |
| Khan^26^ | 2021 | India | NR | 268 | 5 | 49 | 0 | 219 | Biopsy, curettage, hysterectomy | Range 15-70 | Percentage and intensity | Absent staining | Intensity scale | NR |
| Huang M.^27^ | 2013 | USA | R | 24 | 12 | 22 | 0 | 2 | LS group: hysterectomy  Control:NR | NR | Percentage | <10% positive staining | Loss/ presenсe | One |
| Kapucuoglu^28^ | 2007 | Turkey | R | 37 | 2 | 10 | 0 | 27 | Biopsy, curettage | NAEH: SH - 47.8 (35.0–38.0), CH - 48.6 (39.0–55.0) AH/EIN - 50.1 (32.0–64.0) | Percentage and intensity, score 0-300 | Complete loss | Loss/ presenсe | One |
| Lacey^29^ | 2008 | USA | R | 308 | 41 | 73 | 105 | 235 | Biopsy | NR | Null gland | Any null gland | Loss/ presenсe | One |
| Lee^30^ | 2012 | Korea | R | 42 | 15 | 21 | 5 | 21 | NR | Median 47  Range 29 – 77 | Percentage | >5% absent staining in glands | Graded scale | NR |
| Pavlakis^31^ | 2010 | Greece | R | 83 | 38 | 58 | 15 | 25 | Curettage | Range 35 - 67 | Percentage | >80% absent staining in glands | Loss/ presenсe | Three |
| Pieczyńska^32^ | 2011 | Poland | R | 132 | 1 | 16 | 4 | 116 | Curettage | NR | Percentage and intensity, score 0-300 | Complete loss | Loss/ presenсe | NR |
| Rani^33^ | 2019 | India | R | 47 | 9 | 9 | ~~4~~ | 38 | NR | All - 46.73±11.55 (26-79) | Percentage and intensity | <10% positive staining, intensity 0 | Graded scale | NR |
| Rao^34^ | 2011 | India | R-P | 76 | 13 | 13 | 46 | 63 | Curettage, hysterectomy | Range 35 - 55 | Null gland | Any null gland | Graded scale | NR |
| Sarmadi^35^ | 2009 | Iran | R | 29 | 2 | 8 | 0 | 21 | Curettage | NR | Percentage and intensity | < 10% positive staining, intensity 0 | Graded scale | Two |
| Shawana^36^ | 2016 | Pakistan | R | 26 | 4 | 6 | 1 | 20 | Curettage, hysterectomiy | NAEH: SH - 43.88 ± 8.09  CH - 41.90 ± 8.04  AH/EIN: 47.01 ± 8.05 | Percentage and intensity | <10% positive staining, intensity 0 | Graded scale | NR |
| Stoenescu^37^ | 2017 | Romania | P | 96 | 42 | 42 | 54 | 54 | Curettage | All - 52.29 ± 8.14 (With EC) | Intensity scale | Absent staining | Intensity scale | NR |
| Tantbirojn^38^ | 2008 | Thailand | R | 45 | 12 | 20 | 6 | 25 | Curettage | NAEH - 46.68 + 9.92,  AH/EIN - 47.50 + 9.18 | Intensity scale | Absent staining | Intensity scale | NR |
| Upson^39^ | 2012 | USA | R | 112 | 32 | 40 | 39 | 72 | Biopsy | NR | Percentage | >25% absent staining | Loss/presenсe | One |
| **Total WHO1994** |  |  |  | 1345 | 230 | 395 | 279 | 950 |  |  |  |  |  |  |
| **EIN** |  |  |  |  |  |  |  |  |  |  |  |  |  |  |
| Baak^40^ | 2005 | Norway | R | 103 | 14 | 21 | 29 | 82 | Curettage | All - 50.3 (29-71) | Null gland | Any null gland | Loss/ presenсe | NR |
| Mutter^41^ | 2000 | USA | R | 19 | 9 | 12 | 2 | 7 | Biopsy, curettage, hysterectomy | NR | Intensity scale | Absent staining | Intensity scale | Two |
| Mutter^42^ | 2001 | USA | NR | 76 | 22 | 35 | 23 | 41 | Biopsy, curettage | NAEH - 45.2 ± 9.3, AH/EIN- 54.1 ± 7.8 | Null gland | Any null gland | Loss/ presenсe | Two |
| Norimatsu^43^ | 2007 | Japan | R | 70 | 13 | 38 | 4 | 32 | Biopsy,  curettage | NAEH - 51.2 (35-67),  AH/EIN - 53.8 (47-65) | Null gland | Any null gland | Loss/ presenсe | Three |
| Orbo^44^ | 2003 | Norway | R | 68 | 11 | 39 | 3 | 29 | Curettage | All: 48.2 (28–77) | Percentage | <10% positive staining | Loss/ presenсe | One |
| Xiong^45^ | 2010 | China | R | 83 | 9 | 24 | 17 | 59 | NR | NR | Percentage | Complete loss | Graded scale | NR |
| **Total EIN** |  |  |  | 419 | 78 | 169 | 78 | 250 |  |  |  |  |  |  |
| **WHO2014** |  |  |  |  |  |  |  |  |  |  |  |  |  |  |
| Aguilar^46^ | 2023 | USA | R | 195 | 56 | 111 | 13 | 84 | NR | NAEH - 49-77 | Percentage | >10% absent staining in glands | Loss/ presenсe | NR |
| Allithy^47^ | 2022 | Egypt | P | 54 | 0 | 29 | 0 | 25 | Curettage | All: 50.4 (29-72) | Percentage and intensity, score 0-12 | Score 0 (negative) | Graded scale | NR |
| Huang Q.^48^ | 2020 | China | NR | 70 | 25 | 40 | 0 | 30 | NR | NR | Percentage | ≤10% positive staining | Loss/ presenсe | NR |
| Ramzy^49^ | 2022 | Egypt | R | 50 | 8 | 8 | 14 | 42 | NR | All: 47.8 ± 9 | Percentage | ≤50% positive staining | Loss/ presenсe | Two |
| Sanderson^50^ | 2022 | UK | R | 104 | 34 | 51 | 27 | 53 | Biopsy | NAEH - 52.9,  AH/EIN - 52.8 | Null gland | Any null gland | Graded scale | Two |
| Yadav^51^ | 2022 | India | P | 37 | 8 | 10 | 19 | 27 | Resected specimens | All: 45.5±8.6 (35-70) | Percentage and intensity, score 0-16 | Score 0-3 | Loss/ presenсe | NR |
| **Total WHO2014** |  |  |  | 510 | 131 | 249 | 73 | 261 |  |  |  |  |  |  |
| **Total EH** |  |  |  | 2274 | 439 | 813 | 430 | 1461 |  |  |  |  |  |  |

**
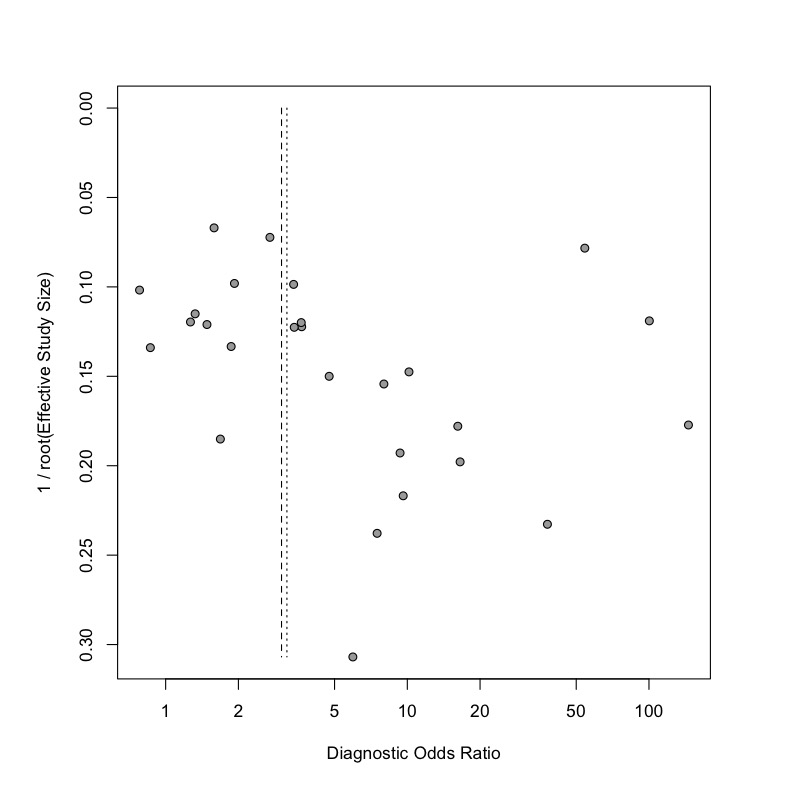
**

**Figure S1**. Funnel plot for publication bias assessment among studies on PTEN loss in the detection AH/EIN.

Test result: t = 1.12, df = 25, p-value = 0.2715

Bias estimate: 7.2517 (standard error = 6.4485); multiplicative residual heterogeneity variance (tau^2 = 134.6992).

**
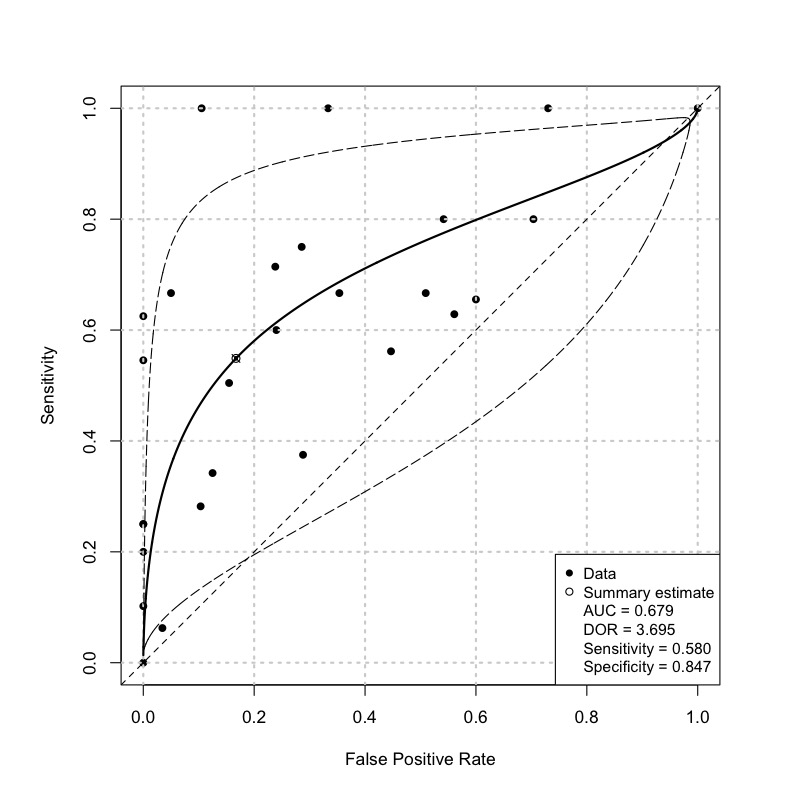
**

**Figure S2.** Summary Receiver Operating Characteristic (SROC) curve for the diagnostic estimates of PTEN loss by immunohistochemical assessment for differentiating AH/EIN from non-atypical endometrial hyperplasia. Solid line represents the SROC curve, white point shows the joint estimates, dashed line represents the 95% confidence region, dotted line represents the 95% prediction region

**Table S3.** Subgroup Analysis

| **Subgroup** | Studies | Sensitivity (95% CI) | Specificity (95% CI) | PosLR (95% CI) | NegLR (95% CI) | DOR (95% CI) | AUC |
| --- | --- | --- | --- | --- | --- | --- | --- |
| **Overall** | **27** | 0.580 (0.408–0.734) | 0.847 (0.67–0.938) | 1.926 (1.510–2.810) | 0.710 (0.583–0.732) | 3.695 (2.501–5.40) | **0.679** |
| **I² (%)** |  | I² = 69.8% | I² = 82.4% | I² = 87.3 % | I² = 72.2 % | I² = 39.3% |  |
| **Classification** |  |  |  |  |  |  |  |
| WHO1994 | 15 | 0.624 (0.362–0.829) | 0.904 (0.573–0.985) | 2.900 (1.46–5.670) | 0.594 (0.46–0.727) | 4.920 (2.28–9.320) | **0.72** |
| I² (%) |  | I² = 75.3% | I² = 84.1% | I² = 94.7 % | I² = 53.8 | I² = 54.5% |  |
| EIN | 6 | 0.489 (0.345–0.635) | 0.729 (0.577–0.842) | 1.720 (1.210–2.490) | 0.732 (0.591–0.873) | 2.400 (1.41–3.83) | **0.586** |
| I² (%) |  | I² = 72.7% | I² = 76.1% | I² = 32.85% | I² = 0.00% | I² = 0.00% |  |
| WHO2014 | 6 | 0.578 (0.159–0.908) | 0.858 (0.438–0.979) | 2.560 (1.260–5.390) | 0.576 (0.454–0.711) | 4.510 (1.87–9.20) | **0.701** |
| I² (%) |  | I² = 20.1% | I² = 83.6% | I² = 78.87%, | I² = 86.40 % | I² = 52.0% |  |
| **Method of PTEN IHC Assessment** |  |  |  |  |  |  |  |
| Percentage and intensity of staining | 9 | 0.282 (0.078–0.648) | 0.992 (0.829–1) | 6.900 (2.460–16.000) | 0.721 (0.521–0.874) | 9.710 (3.260–22.700) | **0.744** |
| I² (%) |  | I² = 63.5% | I² = 83.1% | I² = 64.11 % | I² = 32.49 % | I² = 39.4% |  |
| Percentage of cell stained | 9 | 0.603 (0.468–0.724) | 0.756 (0.579–0.875) | 1.760 (1.400–2.220) | 0.643 (0.527–0.761) | 2.770 (1.910–3.900) | **0.657** |
| I² (%) |  | I² = 72.0% | I² = 71.2% | I² = 58.15% | I² = 62.27% | I² = 48.7% |  |
| Presence of null glands | 6 | 0.641 (0.47–0.782) | 0.55 (0.388–0.703) | 1.360 (1.090–1.770) | 0.731 (0.596–0.885) | 1.890 (1.240–2.770) | **0.576** |
| I² (%) |  | I² = 53.3% | I² = 85.1% | I² = 0.00% | I² = 0.00% | I² = 0.00% |  |
| Intensity of staining | 3 | 0.9 (0.389–0.992) | 0.203 (0.003–0.958) | 1.880 (0.985–6.79) | 0.589 (0.219–1.53) | 4.010 (0.640–13.60) | **0.694** |
| I² (%) |  | I² = 0% | I² = 0% | I² = 70.56% | I² = 0%, | I² = 0% |  |

Area under the receiver operating characteristic curve (AUC), Diagnostic odds ratio (DOR), Positive likelihood ratio (PosLR), Negative likelihood ratio (NegLR)

**Figure S4-S6:** Summary Receiver Operating Characteristic (SROC) curve for the diagnostic estimates of PTEN loss by immunohistochemical assessment for differentiating AH/EIN from non-atypical endometrial hyperplasia for the WHO1994 classification (S3), EIN (S4) and WHO2014 (S5). Solid line represents the SROC curve, white point shows the joint estimates, dashed line represents the 95% confidence region, dotted line represents the 95% prediction region

| 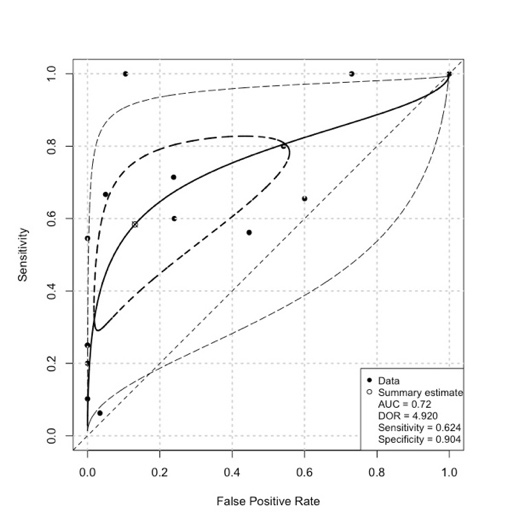 | Figure S3  WHO1994 |
| --- | --- |
| 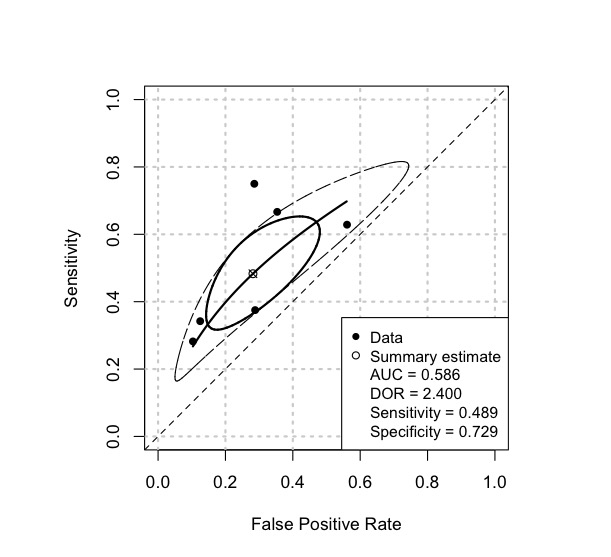 | Figure S4  EIN |
| ~~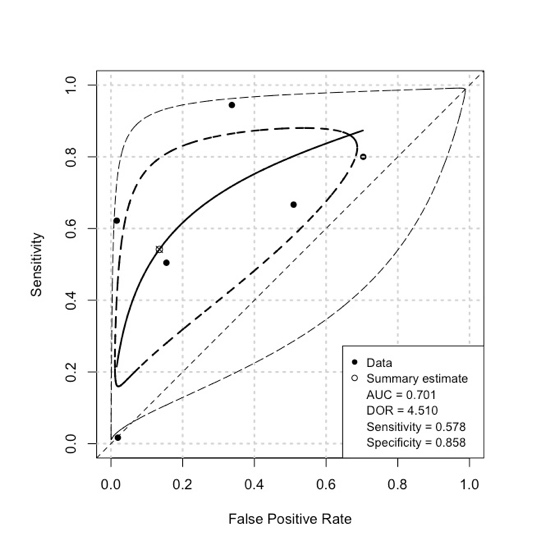~~ | Figure S5  WHO2014 |

Table S4. Characteristics of included studies reporting the diagnostic estimates of combination of different immunohistochemical markers for differentiating AH\EIN and non-atypical EH.

| IHC Marker Combination | First author, year | Total  patients | Non-atypical EH (N) | AH/EIN (N) | Abberrant in non-atypical EH  n/N (%) | Abberrant in AH/EIN  n/N (%) |
| --- | --- | --- | --- | --- | --- | --- |
| PTEN,  β-catenin; | Norimatsu,^43^  2007 | 70 | 32 | 38 | 4/32 (**12.5%)** | 22/38 **(57.9%)** |
| PTEN,  PAX2,  β-catenin; | Aguilar,^46^  2023 | 141 | 40 | 111^1^ | 19/40 (47.5%) | 103/111 (92.8%) |
| PTEN, HAND2, PAX2 (cluster-based) | Sanderson,^50^  2022 | 105 | 54 | 51 | 47/54 (**87.0%)** | 44/51 **(86.3%)** |

AH/EIN = atypical hyperplasia; EH = endometrial hyperplasia;

^1^The 111 AH/EIN cases in Aguilar et al. were from a previously published study (Aguilar M, Chen H, Rivera-Colon G, et al. Reliable identification of endometrial precancers through combined Pax2, β-catenin, and Pten

immunohistochemistry. Am J Surg Pathol. 2022;46:404–414.)

^2^Cluster analysis identified 4 clusters based on PTEN, HAND2, PAX2. 47/54 non-atypical EH cases grouped into the “benign” cluster, while 44/51 AH/EIN cases grouped into “non-benign” clusters.
